# Role of neddylation in diabetes metabolism: potential therapeutic target

**DOI:** 10.1007/s40200-025-01837-9

**Published:** 2026-01-03

**Authors:** Hui Li, Ruiyin Zeng, Jing Liu, Huiwen Wang

**Affiliations:** Department of Orthopedics, Union Hospital, Tongji Medical College, Huazhong University of Science and Technology, Wuhan, 430022 China

**Keywords:** Neddylation, Diabetes mellitus, Glucose metabolism, Insulin resistance, Neddylation inhibitor

## Abstract

**Purpose:**

The study aimed to explore the role of neddylation in diabetes metabolism and its potential as a therapeutic target. Given the increasing global diabetes burden and limitations of current treatments, the research question was to determine how neddylation affects diabetes pathophysiology and if targeting it could offer new treatment strategies.

**Methods:**

The authors gathered data from a comprehensive review of existing research on neddylation in diabetes, including studies on insulin signaling, glucose and lipid metabolism, and related diseases. Experimental models, including diabetic mice and granulosa cells, and pharmacological interventions like MLN4924 were also thoroughly reviewed. By collating and evaluating these sources of information, the authors analyzed the influence of neddylation on diverse molecules and pathways implicated in diabetes.

**Results:**

Neddylation is involved in multiple aspects of diabetes. It affects insulin signaling by modifying insulin receptor substrate (IRS), inositol hexakisphosphate (IP6), phosphoenolpyruvate carboxykinase 1 (PCK1), and other molecules. Inhibiting neddylation with drugs like MLN4924 can reduce hepatic glucose generation, alleviate hyperglycemia, and improve metabolic phenotypes in mouse models. Neddylation is also associated with related diseases such as familial hyperkalemic hypertension, obesity - related metabolic disorders, and nonalcoholic steatohepatitis combined with type 2 diabetes.

**Conclusion:**

Neddylation plays a significant role in glucose metabolism regulation and diabetes pathogenesis. Targeting neddylation, especially with inhibitors like MLN4924, shows promise as a new therapeutic strategy for diabetes. Further research on neddylation in diabetes is needed to solidify its potential in treatment and management.

## Background

Diabetes, as a global chronic disease, has become a significant challenge in the field of public health. Statistics show that there are approximately 537 million diabetes patients worldwide, with Type 2 Diabetes Mellitus (T2DM) accounting for nearly 90% of cases, and this number is continuously growing at an alarming rate, particularly among children and young adults [1]. T2DM not only brings profound physical and mental suffering to patients and their families but also imposes a substantial economic burden on healthcare systems.

The pathogenesis of T2DM is intricate, influenced by both genetic and environmental elements. Its typical features include hyperinsulinemia, insulin resistance, and the dysfunction of pancreatic beta-cells, the latter of which can lead to up to 50% of cell apoptosis [2]. Chronic low-grade inflammation and oxidative stress are recognized as pivotal contributors to the development and progression of T2DM. Pro-inflammatory cytokines, such as TNF-α and IL-6, can interfere with insulin signaling pathways, leading to insulin resistance in peripheral tissues. Concurrently, oxidative stress, characterized by an imbalance between reactive oxygen species (ROS) production and antioxidant defenses, contributes to β-cell dysfunction and apoptosis. The interplay between inflammation, oxidative stress, and metabolic dysregulation creates a vicious cycle that exacerbates the diabetic condition [3–5]. In recent years, factors such as incretin effect, alterations in the colon and its microbiota [6, 7], immune dysregulation, and inflammation [8] have also been considered important pathophysiological factors of T2DM and may emerge as new targets for future treatments. Furthermore, the hyperglycemic state can lead to a series of microvascular and macrovascular complications [9], such as endothelial dysfunction [10], advanced glycation end-product formation [11], hypercoagulability, increased platelet reactivity, and upregulation of sodium-glucose cotransporter-2 (SGLT-2) [12], which are also key targets for diabetes treatment.

While Type 1 Diabetes Mellitus (T1DM) is characterized by autoimmune destruction of pancreatic β-cells and absolute insulin deficiency, Type 2 Diabetes Mellitus (T2DM) arises from insulin resistance and progressive β-cell dysfunction. Insulin resistance is a core pathological feature of T2DM, manifested by the body’s ability to secrete normal levels of insulin but inability to effectively suppress glucose production and regulate blood sugar [13]. This phenomenon involves reduced cellular glucose uptake capacity and inhibition of glucose conversion to fat, hepatic glycogen, or muscle glycogen, ultimately leading to excessive glucose accumulation in the body [14, 15]. Current management of T2DM prioritizes lifestyle modifications and oral antihyperglycemic agents (e.g., metformin, SGLT-2 inhibitors), with insulin therapy reserved for advanced cases or T1DM. But these methods have limitations such as poor treatment efficacy and significant side effects. Therefore, the search for novel therapeutic targets and treatment methods to enhance the quality of life and prognosis of diabetes patients is of paramount importance [16].

**Fig. 1:**
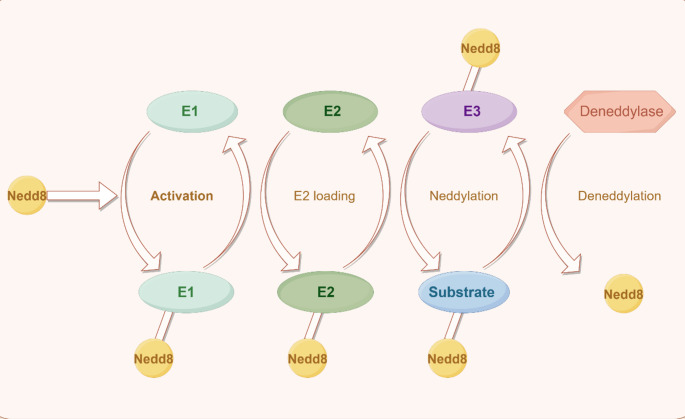
A general description of NEDD8 activation, conjugation, and deneddylation. The NEDD8 precursor is split by C-terminal hydrolases like UCHL3 and NEDP1. E1 NEDD8 activating enzyme (NAE), E2 Nedd8-conjugative enzyme (Ube2M or Ube2F), and E3 NEDD8 ligase are involved in the protein neddylation process. Deneddylase removes NEDD8 from neddylated substrates, releasing it for subsequent neddylation cycles. This figure was drawn in Figdraw

In recent research, a protein post-translational modification process called neddylation has garnered widespread attention. Neddylation encompasses a series of reactions involving E1, E2, and E3 enzymes, attaching the neuronal precursor cell-expressed developmentally downregulated protein 8 (NEDD8) to the lysine residue of substrate proteins, thereby modulating protein stability, subcellular localization, and function. This process is similar to ubiquitination but holds unique physiological significance [17]. Neddylation has been demonstrated to be crucial in diverse physiological functions including heart and embryonic development, synapse formation and maturation, adipogenesis, and the progression of tumors [18–21]. The most representative neddylation substrate is cullin, an essential component of the Cullin-RING E3 ubiquitin ligase complex (CRL). Neddylation of cullin enhances CRL activity, promoting cell cycle progression and cell survival [22]. Moreover, non-cullin proteins are also crucial in neddylation research. These substrates include oncogenes, tumor suppressors, transcription factors, histones and modification enzymes, ribosomal proteins, and signaling transducers [23] (Fig. 1).

The significance of neddylation in metabolic disorders such as diabetes has been increasingly understood in recent years. Studies have shown that neddylation plays a crucial role in regulating insulin signaling pathways and glucose and lipid metabolism [17, 24, 25]. Therefore, therapeutic strategies targeting neddylation may offer new insights for the treatment of diabetes. This article aims to review the role and mechanism of neddylation in diabetes and lipid metabolism and explore its potential as a therapeutic target, with the hope of providing new strategies and directions for the treatment of diabetes.

## Literature search strategy

This narrative review was based on a comprehensive literature search conducted in electronic databases including “Med-line”, “PubMed” and “Scopus”. The search was performed using a combination of the following keywords: “neddylation”, “NEDD8”, “diabetes”, “diabetes mellitus”, “insulin resistance”, “glucose metabolism”, “MLN4924”, “Pevonedistat”. Relevant original research articles and reviews were selected, with a focus on studies elucidating the molecular mechanisms linking neddylation to metabolic disorders. The findings from these studies were synthesized to provide an overview of the field.

## Insulin signal transduction mechanism and regulation of metabolism

Insulin assumes an essential part in sustaining the energy equilibrium of the organism. Upon binding to the insulin receptor (IR) located on the cell membrane, the tyrosine kinase activity of the IR is activated, thereby triggering a series of signal cascade reactions [26]. This process involves the phosphorylation of multiple signal molecules, especially the members of the insulin receptor substrate (IRS) protein family (IRS-1, IRS-2, IRS-3, and IRS-4), which exhibit tissue-specific functional divergence. For instance, IRS-1 primarily mediates metabolic and mitogenic effects in skeletal muscle and liver, whereas IRS-2 dominates glucose homeostasis and pancreatic β-cell function. IRS-3/4 act as negative regulators to prevent excessive PI3K signaling [27].

As a key signal transmitter, the IRS protein, after being phosphorylated, can attract and activate a variety of downstream effector molecules, such as phosphatidylinositol 3-kinase (PI3K). The activation of PI3K further leads to the phosphorylation of Akt (also known as PKB), which is a core molecule in the insulin signaling pathway and participates in the regulation of a variety of cellular physiological processes [28, 29]. For example, after Akt activation, glycogen synthesis can be induced by inhibiting GSK-3; protein synthesis can be carried out through mTOR and downstream components; cell survival can be promoted by inhibiting multiple pro-apoptotic factors (Bad, FOXO transcription factors, GSK-3 and MST1).

The regulatory role of insulin in glucose and lipid metabolism is a crucial component of its biological function [30, 31]. In muscle and adipose tissues, insulin promotes glucose uptake and utilization by stimulating the translocation of GLUT4 vesicles to the plasma membrane. Skeletal muscle cells express a low Km hexokinase isoform, making their glucose uptake highly dependent on insulin-mediated IRS signaling. In contrast, hepatocytes and pancreatic β-cells achieve non-insulin-dependent glucose sensing and metabolic regulation through the expression of GLUT2. Hepatocytes continuously express glucose transporter 2 (GLUT2) at their basolateral membrane, with its transport capacity driven directly by the concentration gradient of glucose inside and outside the cell. Once glucose enters hepatocytes, it is rapidly phosphorylated by glucokinase (GK) into glucose-6-phosphate. This enzyme exhibits high Km characteristics, which synergizes with the kinetic properties of GLUT2 to ensure that the liver initiates glycogen synthesis or glycolysis only when blood glucose levels are significantly elevated. Additionally, β-cells sense blood glucose levels via GLUT2, triggering insulin secretion [32]. Together, GLUT4-mediated insulin-dependent glucose uptake and GLUT2-mediated insulin-independent glucose transport form a dual anchor for blood glucose regulation. This process is crucial for maintaining the stability of blood sugar levels [33].

**Fig. 2:**
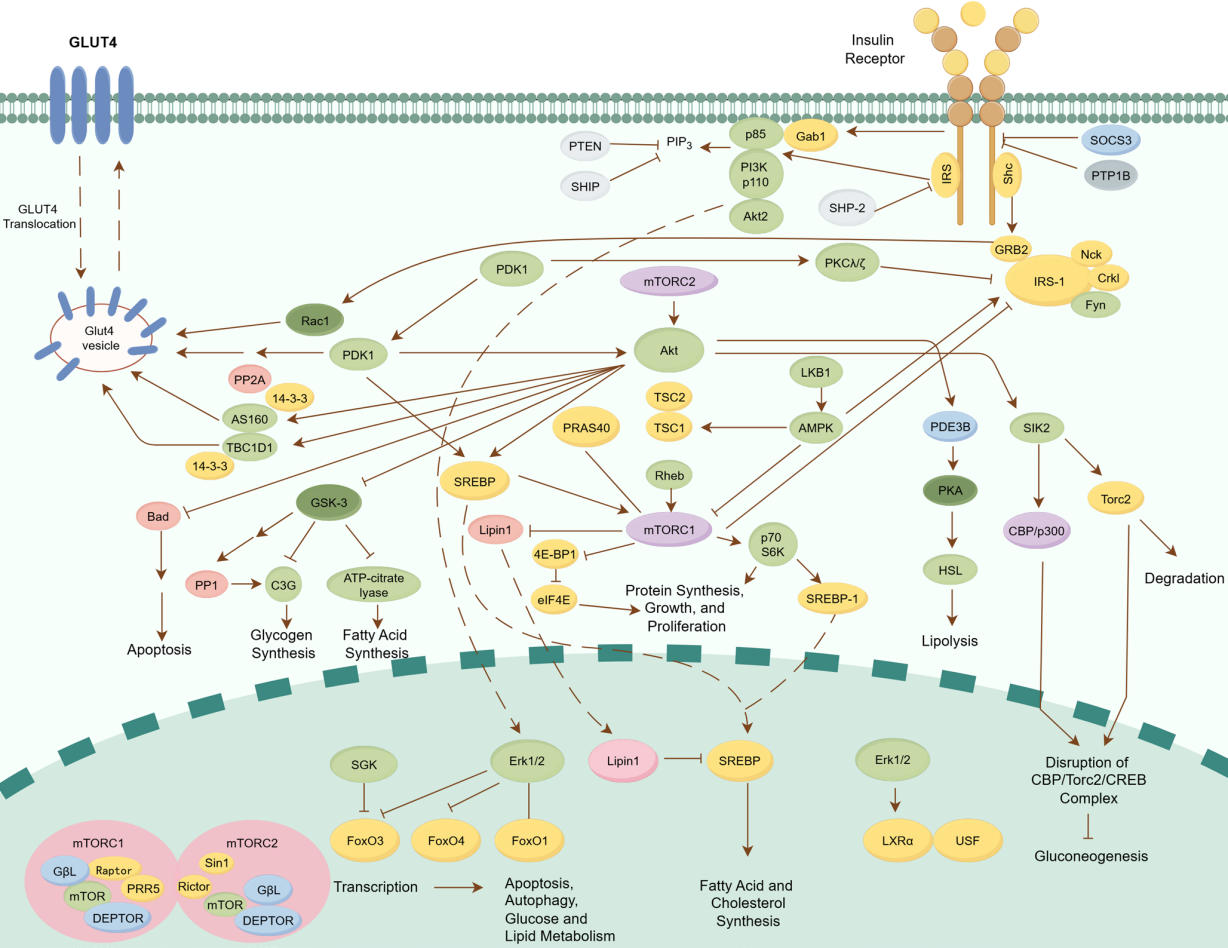
The schematic diagram provides an overview of the insulin signaling pathway. Insulin binds to the IR on the cell membrane, activating its tyrosine kinase activity and initiating a series of signal cascade reactions that involve the phosphorylation of signal molecules such as IRS. Subsequently, IRS attracts and activates downstream effector molecules such as PI3K, leading to the phosphorylation of Akt. Akt plays a role in regulating diverse cellular physiological processes. The insulin signaling pathway regulates glycolipid metabolism in muscle and adipocytes, promotes the uptake and utilization of glucose, and affects fatty acid and cholesterol metabolism by regulating a series of transcription factors and enzymes, maintaining the balance of glycolipid metabolism in the body. Sharp arrows signify stimulatory modifications, blunt arrows represent inhibitory modifications, and dashed lines indicate translocation processes. This figure was drawn in Figdraw

In addition, insulin also affects the synthesis and decomposition of fatty acids, as well as the metabolism of cholesterol, through the regulation of various transcription factors and enzymes. For example, insulin can activate the SREBP transcription factor, thereby promoting the production of cholesterol and fatty acids [34]; at the same time, by activating transcription factors such as USF1 and LXR, insulin can also promote the synthesis of fatty acids [35]. These effects jointly maintain the balance of glycolipid metabolism in the body (Fig. 2).

## Abnormal Neddylation Modification in Diabetes Mellitus: Impact on Insulin Signaling

1. Neddylation and IRS
  As previously stated, the insulin receptor (IR) serves as a pivotal molecule in insulin signaling, activating a cascade of pathways upon binding insulin, thereby promoting glucose uptake and utilization in cells [36]. The aberrant activation of kinases that respond to nutrient and stress stimuli results in the phosphorylation of insulin receptor substrate (IRS) at serine/threonine sites, followed by proteasomal degradation of IRS. This represents a significant underlying factor contributing to hepatic insulin resistance. Several studies have observed significantly higher levels of neddylation in diabetic and hyperglycemic animals compared to healthy individuals [37–39]. A common mechanism involves abnormally activated neddylation of cullin 1 and 3, mediating accelerated and abnormal degradation of IRS proteins in hepatocytes. MLN4924, which serves as an inhibitor of NEDD8-activating enzyme (NAE), is currently undergoing clinical trials for cancer therapeutics [22]. Research has shown that neddylation modification is involved in IRS1 and IRS2-dependent insulin signaling, and inhibition of neddylation with MLN4924, an NAE inhibitor, can rapidly decrease hepatic glucose generationand alleviate hyperglycemia in mice through the activation of IRS1 and IRS2 as well as the PI3K/AKT signaling pathway[39, 40].
2. Neddylation and IP6
  IP6-assisted competitive regulation by COP9 signalosome-Constitutively Photomorphogenic 1 (CSN-COP1) modulates the CRL4-ETV5 proteolytic checkpoint, thus securing insulin secretion stimulated by glucose. COP1 and COP9 signalosome individually function as the substrate receptor and deneddylase for the CRL4 E3 ligase. Heterozygous Csn2WT/K70E mice, which have a partial IP6 binding disruption, are prone to congenital hyperinsulinism and insulin resistance, as studies have uncovered. This condition is caused by amplified Cul4 neddylation, CRL4COP1 E3 ligase assembly, and ETV5 ubiquitination. Hyperglycemia inversely influences the formation of CRL4-CSN and CRL4COP1, fostering ETV5 breakdown. However, neddylation inhibitors such as Pevonedistat/MLN4924 stabilize ETV5 and improve hyperinsulinism and obesity/diabetic phenotypes in these mice [41].
3. Neddylation and PCK1
  The PCK1 gene is a major regulator of gluconeogenesis, encoding a cytosolic enzyme which catalyzes the generation of phosphoenolpyruvate from oxaloacetate, releasing carbon dioxide and GDP [42]. Its expression is controlled by insulin, glucocorticoids, glucagon, cAMP, and diet. PCK1 is considered one of the many genes associated with type 2 diabetes. Studies have found that neddylation in mouse livers is regulated by the availability of nutrients. Inhibiting neddylation in mouse livers leads to a reduction in gluconeogenic capacity and counteracts the hyperglycemic effects of regulatory hormones. This mechanism may involve neddylation of phosphoenolpyruvate carboxykinase 1 (PEPCK, encoded by the PCK1) at three lysine residues - K278, K342, and K387, triggered by fasting or calorie restriction in mice [37].
4. Neddylation and Glycolytic Enzymes
  A study revealed a glucose-induced CRL4COP1-p53 axis that reinforces glucose uptake and glycolysis. More precisely, glucose-dependent CK2 O-GlcNAcylation hinders the phosphorylation of CSN2, a modification necessary for the sequestration of Cullin RING Ligase 4 (CRL4) by the deneddylase CSN. As a result, glucose stimulates the dissociation of CSN and CRL4, enabling the formation of CRL4COP1 E3 ligase. This ligase subsequently aims at p53, lifting the suppression on glycolytic enzymes [43]. Another study on inhibiting neddylation in cancer cells found that MLN4924, a small-molecule inhibitor targeting neddylation, hinders the transition between mitochondrial fission and fusion in breast cancer cells. This inhibition occurs by impeding the ubiquitination and degradation of MFN1. Simultaneously, MLN4924 suppresses the TCA cycle while enhancing mitochondrial OXPHOS. Furthermore, MLN4924 induces the activation of PKM2 by facilitating its tetramerization, consequently enhancing glycolysis [44]. Although this study was conducted in cancer cells, the regulatory role of MLN4924 in mitochondrial function and glycolysis may provide insights into its potential effects on glucose metabolism in diabetic conditions, which requires further validation in diabetic cell or animal models.
5. Neddylation and the KLHL2/RhoBTB1/KLHL3 Axis
  KLHL2, RhoBTB1, and KLHL3 are proteins closely related to protein degradation and cell function regulation. They belong to the Kelch-like proteins (KLHLs) family and are components of the Cullin-RING E3 Ligase Complex (CRL). A study found significantly increased levels of Cullin 3 and its neddylated derivatives in the aortic tissue and heart of diabetic mice. Hyperglycemia and hyperinsulinemia lead to overactivation of neddylation, resulting in the inactivation of KLHL2/RhoBTB1/KLHL3 in aortic tissue and kidneys. This malfunction of the Cullin 3 RING E3 ubiquitin ligase contributes to amplified vasoconstriction and sodium reabsorption in diabetic individuals [45].
6. Neddylation and PTEN
  The PTEN tumor suppressor regulates the PI3K/Akt signaling cascade within the cytoplasm and upholds chromosomal stability in the nucleus [46]. Research has indicated that under conditions of elevated glucose, PTEN interacts with Nedd8, triggering its relocation to the nucleus without compromising its stability. Once neddylated PTEN gathers in the nucleus, it facilitates cellular proliferation and metabolic activities rather than suppressing them. PTEN dephosphorylates the FASN protein, hindering TRIM21-mediated FASN ubiquitination and degradation, thus fostering the synthesis of new fatty acids [47].
7. Neddylation and p62 Protein
  The p62 protein is an adaptor protein between autophagosomes and substrates, acting as a molecular regulator in the process of autophagy. p62 protein plays a key regulatory role in amino acid signaling pathways through its influence on the mTORC1 signaling pathway. Studies have found that p62 is often overexpressed in tumors. The presence of p62 impedes the binding of DCNL1 (also referred to as DCUN1D1) to CUL2, thereby reducing the neddylation of CUL2 and consequently diminishing the activity of the VHL E3 ligase complex. This results in an elevated expression of HIF1α, which subsequently enhances the glycolytic pathway [48].

## Neddylation and granule cell metabolism

A study focusing on sheep granulosa cells found that MLN4924 induced changes in mitochondrial morphology, heightened cellular glucose uptake, and lactic acid production, increased reactive oxygen species (ROS) generation, and promoted glycolysis. These effects collectively altered mitochondrial function and metabolic pathways [49].

**Fig. 3:**
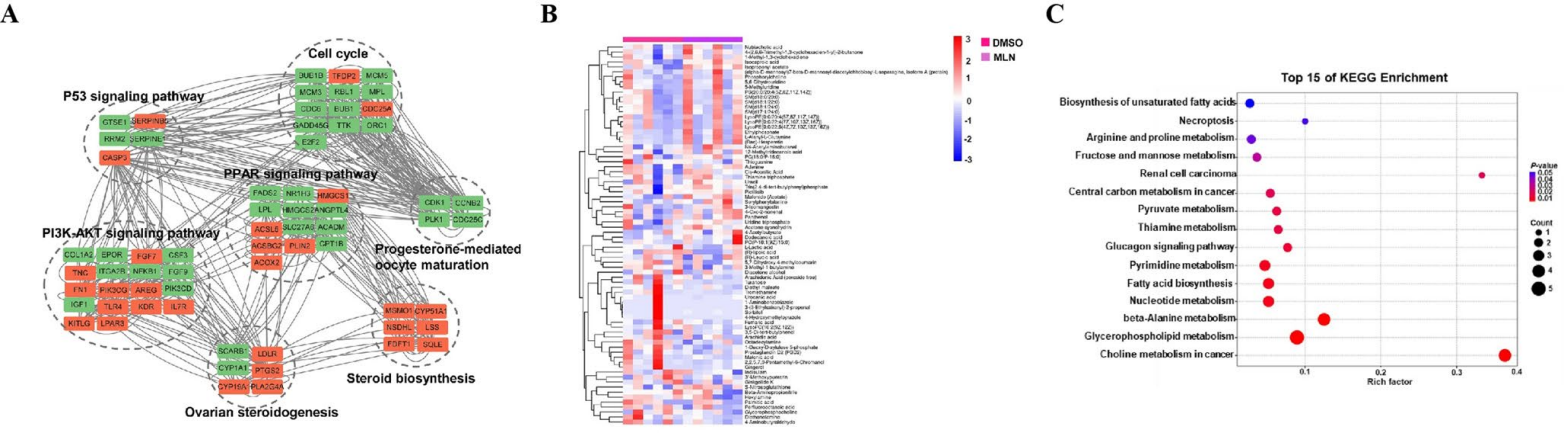
Metabolomics analysis and functional enrichment analysis of DEGs in ovarian granulosa cells treated with DMSO and MLN4924 A. Networks of protein interactions reveal proteins that are differentially expressed within key pathways undergoing notable changes due to MLN4924 treatment. Proteins denoted by orange nodes are upregulated, whereas green nodes signify downregulation B. The heatmap visualizes the relative abundances of metabolites within different group. Each vertical column on the graph corresponds to a specific sample, while horizontal rows depict individual metabolites. Varying colors on the map represent different expression levels C. Significant metabolic differences were identified using statistical analysis via a KEGG pathway enrichment scatter plot. The plot’s x-axis measures the enrichment factor, the y-axis lists various pathways, the point color corresponds to the P-value, and the point size reflects the enriched count. The KEGG pathway enrichment analysis presented in this figure was performed by the authors of the original study [50] Reproduced under the terms of a Creative Commons Attribution 4.0 International License [50]. Copyright 2021, The Authors

Similarly, transcriptional and metabolomic profiling of rabbit granulosa cells treated with MLN4924 identified 563 upregulated and 910 downregulated differentially expressed genes (DEGs), which were enriched in cancer, cell cycle, PI3K-AKT, progesterone-mediated oocyte maturation, and PPAR signaling pathways. Additionally, differential metabolite pathway enrichment highlighted 15 significant pathways, including glycerophospholipid metabolism, fatty acid biosynthesis, unsaturated fatty acid biosynthesis, and fructose and mannose metabolism. Thus, MLN4924 influences lipid and carbohydrate metabolism by modulating metabolite secretion and associated pathways [50] (Fig. 3).

## Neddylation in Metabolic Disorders Related to Diabetes

1. Neddylation and Obesity-Related Metabolic Disorders
  Obesity is one of the major risk factors for type 2 diabetes (T2DM), and obesity-related metabolic disorders (e.g., dyslipidemia, insulin resistance) are key contributors to T2DM development [51, 52]. Neddylation plays a critical role in lipid metabolism, which is closely associated with obesity and subsequent T2DM. The post-translational modification (neddylation) of PPARγ is crucial for adipogenesis. Studies have found that NEDD8 may participate in the stabilization of PPARγ2 by competing with ubiquitination, thereby promoting the transcription of genes that are crucial for lipid accumulation and adipogenesis[21]. More specifically, during adipogenesis, NEDD8 is induced to be highly expressed in preadipocytes and tightly binds to PPARγ, thereby enhancing the stability of PPARγ. Furthermore, the application of the inhibitor MLN4924 has effectively inhibited the symptoms of obesity and glucose intolerance triggered by a high-fat diet in mice. Another study focused on the key role of UBE2M in macrophage-induced obesity-related inflammation. Obesity and other symptoms were significantly alleviated in UBE2M-deficient mice. The mechanism involves UBE2M deficiency inhibiting the neddylation of E3 ubiquitin ligase TRIM21, resulting in diminished recruitment and ubiquitination-mediated degradation of VHL**,** which in turn act on HIF-1α-induced IL-1β production. The study also proposed the use of red blood cell exosomes loaded with Trim21 antisense oligonucleotides to target macrophage TRIM21, inhibiting obesity-induced inflammation and associated metabolic disorders [53]. Moreover, neddylated cullin 3 (Cul3) plays a significant role in both adipogenesis and hepatocellular carcinoma. During the adipogenesis process, once Cul3 is activated by neddylation, it binds to Rab18, promoting its ubiquitination and degradation, thereby influencing the formation of lipid droplets and the stability of RhoA. In hepatocellular carcinoma, elevated Cul3 expression correlates with poor patient prognosis and advances liver cancer development by disrupting the assembly of the SKP1-Cullin-1-F-box protein (SCF) and lipid accumulation. The inhibition of neddyation by using MLN4924 prevents the formation of lipid droplets (LD), lipid storage organelles, and adipogenesis markers [54, 55].
2. Neddylation and Nonalcoholic Steatohepatitis (NASH) Combined with Type 2 Diabetes
  Nonalcoholic steatohepatitis (NASH) is a common comorbidity of T2DM, with shared pathological mechanisms such as insulin resistance and chronic inflammation [56, 57]. In individuals suffering from steatohepatitis comorbid with type 2 diabetes, the downregulation of AGEs scavenger receptor AGER1 and the induction of the proinflammatory receptor RAGE are associated with increased liver fibrosis and inflammation. The dysregulation of NRF2, caused by neddylation of cullin 3, is a critical factor in the downregulation of AGER1. Restoring NRF2 levels can reverse these effects and improve liver health in mouse models [58].

## Exploring neddylation as a potential therapeutic target

MLN4924, as a NEDD8-activating enzyme (NAE) inhibitor, exhibits significant hypoglycemic effects in a diabetic mouse model by blocking the neddylation modification of substrate proteins. This drug can enhance liver sensitivity to insulin, reduce hepatic glycogen output, and effectively lower blood glucose levels. Moreover, MLN4924 also shows potential to improve the immune microenvironment of diabetic wounds and promote wound healing, providing a new strategy for the treatment of diabetes [39, 59].

However, it is important to note that all current evidence supporting the hypoglycemic potential of MLN4924 comes from animal studies, and there is a lack of clinical trials or human experimental data verifying its efficacy and safety in diabetic patients. Metformin’s antihyperglycemic effects are well-established in humans through inhibition of both PEPCK and G6Pase [60, 61], whereas the translation of MLN4924’s animal study results to human diabetes treatment remains unproven. Future research should prioritize conducting well-designed studies to evaluate the feasibility of MLN4924 as an antidiabetic agent in humans.

Beyond its unproven translational value in diabetes, MLN4924 is currently undergoing Phase I/II/III clinical trials for cancer treatment—including acute myeloid leukemia, melanoma, lymphoma, and solid tumors—while its application in diabetes management faces several challenges. In Phase I trials, adverse reactions were noted, including liver toxicity characterized by abnormal liver function tests, which limits its clinical applicability. Other side effects such as gastrointestinal reactions have also been reported. Furthermore, when combined with other chemotherapy agents in certain trials, MLN4924 may increase the incidence or alter the manifestation of adverse effects, such as exacerbated bone marrow suppression [62–64].

These adverse reactions underscore the need for a careful assessment of safety in the further development and application of MLN4924. Optimizing dosing regimens and exploring appropriate dosage frequencies are essential to balance efficacy and safety. Additionally, there is a pressing need for the continued development of safer and more effective NAE inhibitors to reduce the incidence and severity of side effects while improving patient tolerability and therapeutic outcomes [65].

Beyond MLN4924, targeting other critical nodes within the neddylation pathway also shows promise. New inhibitors are actively being explored for clinical applications, primarily in oncology. For instance, TAS4464 encountered challenges due to liver toxicity in early clinical trials; however, its high potency and selectivity provide valuable insights for future optimization and clinical development [66]. Moreover, inhibitors that disrupt the DCN1-UBC12 interaction may play significant roles in specific diseases or combination therapies due to their selective modulation of specific cullin proteins [67]. But most of these inhibitors remain in preclinical research stages.

## Limitations and future perspectives

Current research on Neddylation in diabetes metabolism presents several areas that require further exploration. Firstly, although substantial evidence linking Neddylation modification to diabetes has been accumulated from animal models and in vitro studies, the translation of these findings to human clinical applications remains uncertain due to interspecies differences in metabolic mechanisms and regulatory networks. There is currently a lack of direct clinical data validating the specific effects of Neddylation inhibitors on diabetes pathogenesis in humans. Secondly, the regulatory mechanisms of Neddylation in different subtypes of diabetes are not yet fully understood. Type 1 diabetes is characterized by autoimmune-mediated β-cell damage, while type 2 diabetes primarily involves insulin resistance. It remains unclear whether Neddylation plays a role in the inflammatory response and apoptosis of β-cells in type 1 diabetes or how it specifically affects hepatic insulin resistance and adipocyte function in type 2 diabetes. Moreover, inhibitors such as MLN4924, which target the Neddylation pathway, may interfere with other physiological processes dependent on cullin-RING ubiquitin ligases, potentially leading to off-target effects. The long-term impact of such drugs on human metabolism, including lipid and uric acid levels, has yet to be systematically evaluated through large-scale clinical studies.

To overcome these limitations, future studies should adopt a multi-faceted approach. Precision medicine demands the development of targeted Neddylation inhibitors. For example, by constructing drug carriers specific to the PCK1-PEPCK pathway, we can precisely regulate hepatic gluconeogenesis while minimizing systemic side effects and enhancing drug safety and efficacy. Furthermore, leveraging advancements in artificial intelligence and multi-omics technologies can facilitate the integration of machine learning algorithms with transcriptomic, proteomic, and metabolomic data to construct a more robust model of the Neddylation regulatory network [68]. This approach could aid in screening for highly selective small molecules and predicting potential off-target effects to inform drug optimization efforts.

## Data Availability

Not applicable.

## Abbreviations

T2DM: Type 2 Diabetes Mellitus
CRL: Cullin-RING E3 ubiquitin ligase
IR: insulin receptor
IRS: insulin receptor substrate
IP6: inositol hexakisphosphate
PI3K: phosphatidylinositol 3-kinase
NAE: NEDD8 activating enzyme
Ube2M or Ube2F: Nedd8-conjugative enzyme
NEDD8: neuronal precursor cell-expressed developmentally downregulated protein 8
SGLT-2: sodium-glucose cotransporter-2
PCK1: phosphoenolpyruvate carboxykinase 1
KLHLs: Kelch-like proteins
ROS: reactive oxygen species
DEGs: differentially expressed genes
FHHt: familial hyperkalemic hypertension
SCF: SKP1-Cullin-1-F-box protein
COP: constitutive photomorphogenesis
CSN: COP9 signalosome
